# Supplementary material for: Relative contribution of diet and physical activity to increased adiposity among rural to urban migrants in India: A cross-sectional study
Source: PLoS Med. 2020 Aug 7;17(8):e1003234. doi: 10.1371/journal.pmed.1003234 (PMC7413404; doi:10.1371/journal.pmed.1003234)
Supplement: S1 Text — (DOC) [file pmed.1003234.s002.doc]

**INDIAN MIGRANT STUDY**

(Funded by the Wellcome Trust, U.K)

**CLINIC QUESTIONNAIRE**

**(Apply study id label here)**

**(Apply biochemistry id label here)**

**Subject type:**

[1=Factory worker; 2=Spouse of factory worker; 3=Relative of factory

worker; 4=Relative of spouse]

**Factory worker info:**

Name: __________________________

Age: _____________________ years

Factory dept: _____________________

Factory ID: ______________________

Supported by

**ALL INDIA INSTITUTE OF MEDICAL SCIENCES, NEW DELHI**

**CENTRE FOR CHRONIC DISEASE CONTROL, NEW DELHI**

&

**UNIVERSITY OF BRISTOL, U.K.**

**SECTION A: Clinic questionnaire (CQ)**

|  | **Summary sheet (to be completed at time of reimbursement)** | | | | | | | | | |
| --- | --- | --- | --- | --- | --- | --- | --- | --- | --- | --- |
|  | **Component completed** | | | | | | | | | |
| **1.1** | (a) Consent form | (b) Clinic quest. | | | (c) Diet quest. | | | | (d) Medical exam. | |
|  | [1=Yes; 2=No] | [1=Yes; 2=No] | | | [1=Yes; 2=No] | | | | [1=Yes; 2=No] | |
|  | Reimbursement | | |  | | | | | | |
| **1.2** | Reimbursement given | | | [1=Yes; 2=No] | | | | | | |
| **1.3** | Identity proof taken | | | [1=Yes; 2=No] | | | | | | |
|  | Subject recall | | |  | | | | | | |
| **1.4** | Subject needs to be recalled | | | [1=Yes; 2=No] | | | | | | |
| **1.5** | Reason for recall | | | [1=Repeatability study; 2=Incomplete study; 3=Both] | | | | | | |
| **1.6** | If yes, is the subject willing to return? | | | | | [1=Yes; 2=No; 3=Undecided] | | | | |
| **1.7** | If undecided, date status will be reviewed: | | | | | __ __/__ __/__ __ [DD/MM/YY] | | | | |
| **1.8** | **If recalled, clinic visit details** | | | | | | | | | |
|  | (a) Start date of period  [DD/MM/YY] | | (b) End date of period  [DD/MM/YY] | | | | (c) Venue  [1=Factory; 2=Camp] | (d) Travel  [1=Self; 2=Team] | | (e) Outcome  [1=Yes; 2=No] |
|  | __ __/__ __/__ __ | | __ __/__ __/__ __ | | | |  |  | |  |
|  | __ __/__ __/__ __ | | __ __/__ __/__ __ | | | |  |  | |  |
|  | __ __/__ __/__ __ | | __ __/__ __/__ __ | | | |  |  | |  |
| **1.9** | **Summary sheet notes** | |  | | | | | | | |
|  |  | | | | | | | | | |

|  | **Interview details** |  | |
| --- | --- | --- | --- |
| **2.1** | Date of quest. completion | **___ ___ / ___ ___ / ___ ___** [DD/MM/YY] | |
| **2.2** | Time of quest. completion | :  [Hours: minutes; 24-hour clock] | |
| **2.3** | Interviewer code |  | |
| **2.4** | Interviewer initials |  | |
|  | *First of all I would like to collect some details about you and where you live at present* | | |
|  | **Contact details** |  | |
| **3.1** | Family name | **___________________________** [Surname] | |
| **3.2** | First name/middle name | ___________________________[Forename/other name] | |
| **3.3** | Current house address (if any)  [House No./Street/Locality] | ___________________________________________ | |
| **3.4** | Place name | ______________________ [Name of Village/Town/City] | |
| **3.5** | PIN Code |  | |
| **3.6** | Sub-district | __________________________ [Tehsil/Taluk/Mandal] | |
| **3.7** | District | ___________________________ | |
| **3.8** | Nearest railway station | ___________________________ | |
| **3.9** | Nearest big town | __________________________ [In case of village only] | |
| **3.10** | State | __________________________ [Name of country if abroad] | |
| **3.11** | Type of place | [1=Village; 2=Town; 3=Small city; 4=Large city] | |
| **3.12** | Travelling by road or rail, total average journey time between this place and the industry | | [In completed hours] |
| **3.13** | Census code |  | |
| **3.14** | Home telephone number (landline) | ()  [Area code] [Phone number] | |
| **3.15** | Mobile number |  | |
| **3.16** | Were you born and lived here all your life? | | [1=Yes; 2=No] |

| **4.1** | ABOUT YOUR BIRTHPLACE |
| --- | --- |
|  | (a) Place name________________________ (b) Sub-district_______________________ (c) District________________________ |
|  | (d) Nearest railway station____________________ (e) Nearest town____________________ (f) State/UT__________________________ |
|  | (g) Age at leaving:  [Yrs] (h) Type of place:  [1=V; 2=T; 3=SC; 4=LC] (i) Census code: |
|  | **Between your birthplace and current place of residence, if you have lived anywhere for longer than a year, please list all such places in order up to but not including the current residence** (DO NOT include moves within the same place i.e. village/town/city). Enter age at leaving the place in completed years (enter 00 for age less than a year). Type of place relates to at THAT TIME. WRITE IN BLOCK CAPITALS. |
| **4.2** | (a) Place name________________________ (b) Sub-district_______________________ (c) District________________________ |
|  | (d) Nearest railway station____________________ (e) Nearest town____________________ (f) State/UT__________________________ |
|  | (g) Age at leaving:  [Yrs] (h) Type of place:  [1=V; 2=T; 3=SC; 4=LC] (i) Census code: |
|  | (a) Place name________________________ (b) Sub-district_______________________ (c) District________________________ |
|  | (d) Nearest railway station____________________ (e) Nearest town____________________ (f) State/UT__________________________ |
|  | (g) Age at leaving:  [Yrs] (h) Type of place:  [1=V; 2=T; 3=SC; 4=LC] (i) Census code: |
|  | (a) Place name________________________ (b) Sub-district_______________________ (c) District________________________ |
|  | (d) Nearest railway station____________________ (e) Nearest town____________________ (f) State/UT__________________________ |
|  | (g) Age at leaving:  [Yrs] (h) Type of place:  [1=V; 2=T; 3=SC; 4=LC] (i) Census code: |
|  | (a) Place name________________________ (b) Sub-district_______________________ (c) District________________________ |
|  | (d) Nearest railway station____________________ (e) Nearest town____________________ (f) State/UT__________________________ |
|  | (g) Age at leaving:  [Yrs] (h) Type of place:  [1=V; 2=T; 3=SC; 4=LC] (i) Census code: |

|  | (a) Place name________________________ (b) Sub-district_______________________ (c) District________________________ |
| --- | --- |
|  | (d) Nearest railway station____________________ (e) Nearest town____________________ (f) State/UT__________________________ |
|  | (g) Age at leaving:  [Yrs] (h) Type of place:  [1=V; 2=T; 3=SC; 4=LC] (i) Census code: |
|  | (a) Place name________________________ (b) Sub-district_______________________ (c) District________________________ |
|  | (d) Nearest railway station____________________ (e) Nearest town____________________ (f) State/UT__________________________ |
|  | (g) Age at leaving:  [Yrs] (h) Type of place:  [1=V; 2=T; 3=SC; 4=LC] (i) Census code: |
|  | (a) Place name________________________ (b) Sub-district_______________________ (c) District________________________ |
|  | (d) Nearest railway station____________________ (e) Nearest town____________________ (f) State/UT__________________________ |
|  | (g) Age at leaving:  [Yrs] (h) Type of place:  [1=V; 2=T; 3=SC; 4=LC] (i) Census code: |
|  | (a) Place name________________________ (b) Sub-district_______________________ (c) District________________________ |
|  | (d) Nearest railway station____________________ (e) Nearest town____________________ (f) State/UT__________________________ |
|  | (g) Age at leaving:  [Yrs] (h) Type of place:  [1=V; 2=T; 3=SC; 4=LC] (i) Census code: |
|  | (a) Place name________________________ (b) Sub-district_______________________ (c) District________________________ |
|  | (d) Nearest railway station____________________ (e) Nearest town____________________ (f) State/UT__________________________ |
|  | (g) Age at leaving:  [Yrs] (h) Type of place:  [1=V; 2=T; 3=SC; 4=LC] (i) Census code: |
|  | (a) Place name________________________ (b) Sub-district_______________________ (c) District________________________ |
|  | (d) Nearest railway station____________________ (e) Nearest town____________________ (f) State/UT__________________________ |
|  | (g) Age at leaving:  [Yrs] (h) Type of place:  [1=V; 2=T; 3=SC; 4=LC] (i) Census code: |

|  | *Now I would like to collect some personal information about you* | | | | | | | | | | | |
| --- | --- | --- | --- | --- | --- | --- | --- | --- | --- | --- | --- | --- |
|  | Personal details | |  | | | | | | | | | |
| **5.1** | Age last birthday | | [In completed years] | | | | | | | | | |
| **5.2** | Date of birth | | [DD] | | | | | | | | | |
| **5.3** | Month of birth | | [MM] | | | | | | | | | |
| **5.4** | Year of birth | | 19  [YY] | | | | | | | | | |
| **5.5** | Sex | | [1=Male; 2=Female] | | | | | | | | | |
| **5.6** | (a) How many siblings (alive) do you have? | | | | | [Enter 00 if None] | | | | | | |
|  | (b) Of these, how many are older than you? | | | | | [Enter 00 if None] | | | | | | |
| **5.7** | Current marital status | | [1=Never married; 2=Married; 3=Widow/widower;  4=Separated/divorced] | | | | | | | | | |
| **5.8** | If ever married: | |  | | | | | | | | | |
|  | (a) How old when you started living with your spouse after your marriage (first)? | | | | | | [Age in completed years] | | | | | |
|  | (b) Does your spouse normally live with you now? | | | | | | [1=Yes; 2=No] | | | | | |
|  | How many (live) children do you have? | | | | | | | | | | | |
|  | (c) Boys:  [Enter 00 for None] | | | | | (d) Girls:  [Enter 00 for None] | | | | | | |
| **5.9** | What religion do you follow? | | [1=Hinduism; 2=Islam; 3=Sikhism; 4=Christianity;  5=Jainism; 6=Buddhism; 7=Other; 8=None] | | | | | | | | | |
| **5.10** | People from different castes cook their food differently, which can affect their health. Do you mind telling the name of your caste/tribe? If not,  What is the name of your caste/tribe? ________________________________________________ | | | | | | | | | | | |
| **5.11** | Is this a scheduled caste/tribe/other backward caste? | | | | | [1=Scheduled caste; 2=Scheduled tribe;  3=Other backward class; 4=None of them] | | | | | | |
| **5.12** | (a) What is your mother tongue? | |  | | | | | | | | | |
|  | [1=Assamese; 2=Bengali; 3=Gujarati; 4=Hindi; 5=Kannada; 6=Kashmiri; 7=Konkani; 8=Maithili; 9=Malayalam; 10=Marathi; 11=Oriya; 12=Punjabi; 13=Sindhi; 14=Tamil; 15=Telugu; 16=Urdu; 17=Other, specify below] | | | | | | | | | | | |
|  | (b) If other, specify | | | | | __________________________________ | | | | | | |
|  | Primary occupation | | | | |  | | | | | | |
| **5.13** | (a) Respondent: | | | | | (b) Spouse (if married): | | | | | | |
|  | [1=At home doing housework; 2=Unemployed, not seeking work (student/training/retired/disabled; 3=Unemployed, seeking work; 4=Unskilled manual; 5=Semi-skilled manual; 6=Skilled manual; 7=Skilled non-manual; 8=Semi-Professional; 9=Professional] | | | | | | | | | | | |
| **5.14** | Briefly describe your job: ______________________________________________  ______________________________________________________________ | | | | | | | | | | | |
|  | Highest educational level attained | | | | |  | | | | | | |
| **5.15** | (a) Respondent: | | | | | (b) Spouse (if married): | | | | | | |
|  | [1=Illiterate; 2=Literate, no formal education; 3=Up to primary school (class IV); 4=Secondary school (ITI course, class X/XII, Intermediate); 5=Graduate (BA, BSc, BCom, Diploma); 6=Professional degree/postgraduate (MA, MSc, MBBS, MSW, BTech, PhD)] | | | | | | | | | | | |
|  | *Now I am going to ask you some questions about your household* | | | | | | | | | | | |
|  | **Current household circumstances** | | | | |  | | | | | | |
| **6.1** | What kind of household do you currently live in? | | | | |  | | | | | | |
|  | [1=Single; 2=Hostel; 3=Nuclear family (married couple & offspring); 4=Extended family (two related married couples of different generations (i.e. married couple with one of the parents); 5=Joint family (two related married couples from same generation (i.e. two married siblings); 6=Joint-extended; 7=Any other] | | | | | | | | | | | |
| **6.2** | (a) Including yourself, how many people normally live in your household? | | | | | | | | | |  | |
|  | (b) Of these, how many are aged 18 years or above? | | | | | | | | | |  | |
|  | (c) Of those aged 18 years or above, how many are literate (can read and write)? | | | | | | | | | |  | |
| **6.3** | How many rooms are there in your household? (count all rooms including kitchen, bathroom, etc) | | | | | | | | | |  | |
| **6.4** | What is the material used in the construction of the house? | | | | | | | | | |  | |
|  | [1=Kutcha (made from mud, thatch, or other low quality material); 2=Semi-pucca (partly low quality and high quality material); 3=Pucca (high quality material used throughout including roof, walls, floor)] | | | | | | | | | | | |
| **6.5** | What is the main source of lighting for your household? | | | | | | | [1=Electricity; 2=Kerosene; 3=Gas;  4=Oil; 5=Other] | | | | |
| **6.6** | What is the main source of drinking water for members of your household? | | | | | | | [1=Pipe, hand pump or well (in  residence/yard/plot); 2=Pipe, hand  pump or well (public); 3=Other] | | | | |
| **6.7** | What kind of toilet facility does the household have? | | | | | | |  | | | | |
|  | [1=Own flush toilet; 2=Shared flush toilet; 3=Public flush toilet; 4=Own pit toilet/latrine; 5=Shared pit toilet/latrine; 6=Public pit toilet/latrine; 7=No facility/field/bush; 8=Other] | | | | | | | | | | | |
| **6.8** | Does this household own any agricultural land? | | | | | | | [1=Yes; 2=No] | | | | |
| **6.9** | Do you collect rations from a ration card? | | | | | | | [1=Yes; 2=No] | | | | |
| **6.10** | **Does the household own any of the following:** | | | | | | |  | | | | |
|  | (a) Clock/Watch | | | [1=Yes; 2=No] | | | | | | | | |
|  | (b) Radio/Transistor | | | [1=Yes; 2=No] | | | | | | | | |
|  | (c) Television | | | [1=Yes; 2=No] | | | | | | | | |
|  | (d) Bicycle | | | [1=Yes; 2=No] | | | | | | | | |
|  | (e) Motorcycle/scooter/moped | | | [1=Yes; 2=No] | | | | | | | | |
|  | (f) Car | | | [1=Yes; 2=No] | | | | | | | | |
|  | (g) Tractor | | | [1=Yes; 2=No] | | | | | | | | |
|  | (h) Refrigerator | | | [1=Yes; 2=No] | | | | | | | | |
|  | (i) Telephone | | | [1=Yes; 2=No] | | | | | | | | |
|  | *Now thinking back to when you were a child, say 10-12 years old, please answer the following questions about the household where you lived at that time* | | | | | | | | | | | |
|  | **Household circumstances in childhood (at age 10-12 years)** | | | | | | | | | | | |
| **7.1** | What was your father’s occupation at the time? | | | | | | |  | | | | |
|  | [1=At home doing housework; 2=Unemployed, not seeking work (student/training/retired/disabled; 3=Unemployed, seeking work; 4=Unskilled manual; 5=Semi-skilled manual; 6=Skilled manual; 7=Skilled non-manual; 8=Semi-Professional; 9=Professional] | | | | | | | | | | | |
| **7.2** | What was the highest educational level attained by your mother? | | | | | | |  | | | | |
|  | [1=Illiterate; 2=Literate, no formal education; 3=Up to primary school (class IV); 4=Secondary school (ITI course, class X/XII, Intermediate); 5=Graduate (BA, BSc, BCom, Diploma); 6=Professional degree/postgraduate (MA, MSc, MBBS, MSW, BTech, PhD)] | | | | | | | | | | | |
| **7.3** | Were there any literate (can read and write) adults (aged 18 years or above) in your household? | | | | | | | [1=Yes; 2=No] | | | | |
| **7.4** | What was the material used in the construction of the house? | | | | | | | | | |  | |
|  | [1=Kutcha (made from mud, thatch, or other low quality material); 2=Semi-pucca (partly low quality and high quality material); 3=Pucca (high quality material used throughout including roof, walls, floor)] | | | | | | | | | | | |
| **7.5** | What was the main source of lighting for your household? | | | | | | | [1=Electricity; 2=Kerosene; 3=Gas;  4=Oil; 5=Other] | | | | |
| **7.6** | What was the main source of drinking water for members of your household? | | | | | | | [1=Pipe, hand pump or well (in  residence/yard/plot); 2=Pipe, hand  pump or well (public); 3=Other] | | | | |
| **7.7** | What kind of toilet facility did the household have? | | | | | | |  | | | | |
|  | [1=Own flush toilet; 2=Shared flush toilet; 3=Public flush toilet; 4=Own pit toilet/latrine; 5=Shared pit toilet/latrine; 6=Public pit toilet/latrine; 7=No facility/field/bush; 8=Other] | | | | | | | | | | | |
| **7.8** | Did the household own any agricultural land? | | | | | | | [1=Yes; 2=No] | | | | |
| **7.9** | **Did the household own any of the following:** | | | | | | |  | | | | |
|  | (a) Clock/Watch | | | [1=Yes; 2=No] | | | | | | | | |
|  | (b) Radio/Transistor | | | [1=Yes; 2=No] | | | | | | | | |
|  | (c) Television | | | [1=Yes; 2=No] | | | | | | | | |
|  | (d) Bicycle | | | [1=Yes; 2=No] | | | | | | | | |
|  | (e) Motorcycle/scooter/moped | | | [1=Yes; 2=No] | | | | | | | | |
|  | (f) Refrigerator | | | [1=Yes; 2=No] | | | | | | | | |
|  | *Now one question about your body size when you were around 10-12 years old*. | | | | | | | | | | | |
| **7.10** | What were you like then? | | |  | | | | | | | | |
|  | [1=Very thin;2=Thinner than average; 3=Average; 4=Fatter than average; 5=Very fat] | | | | | | | | | | | |
|  | *One* final *question about your housing circumstances, now or anytime in the past. Do you know what a slum looks like? If yes:*  *(if no, please explain the definition to the subject and then ask the question below)* | | | | | | | | | | | |
| **7.11** | Have you ever lived in a slum for longer than 6 months? | | | | | | | |  | | | |
|  | [1=No, never; 2=Used to but not anymore (moved out over 6 months ago); 3=Yes, and still do (anytime in the last 6 months)] | | | | | | | | | | | |
|  | *Now I will ask you a few questions about your health and lifestyle* | | | | | | | | | | | |
|  | **Health and lifestyle** | | | | |  | | | | | | |
| **8.1** | Have your ever used tobacco on a DAILY basis? | | | | | | | | | | | |
|  | (a) Smoked (e.g. Cigarette/ Beedi/ Cigar/ Pipe/ Hukka/ Chillum) | | | | | | | |  | | | |
|  | (b) Chewed (e.g. Tobacco/ Paan masala/ Zarda/Khaini) | | | | | | | |  | | | |
|  | (c) Snuffed | | | | | | | |  | | | |
|  | [1=No, never; 2=Yes, but don’t anymore (stopped over 6 months ago); 3=Yes, and still do (anytime in the last 6 months)] | | | | | | | | | | | |
| **8.2** | Have you ever consumed alcoholic beverages regularly (i.e. at least 10 days a month)? | | | | | | | |  | | | |
|  | [1=No, never; 2=Yes, but don’t anymore (stopped over 6 months ago); 3=Yes, and still do (anytime in the last 6 months)] | | | | | | | | | | | |
| **8.3** | Compared to others of your age, would you say your health over the last 12 months has been: | | | | | | | [1=Very good; 2=Good;  3=Average; 4=Poor; 5=Very poor] | | | | |
|  | (a) In your knowledge, have you ever suffered from any of the following conditions? THIS NEED NOT BE DOCTOR DIAGNOSED. | | | | | | | (b) If yes, age when known (otherwise leave blank) | | | | |
| **8.4** | High blood pressure | [1=Yes; 2=No] | | | | | | [Age in completed years] | | | | |
| **8.5** | Heart disease | [1=Yes; 2=No] | | | | | | [Age in completed years] | | | | |
| **8.6** | Diabetes (high blood sugar) | [1=Yes; 2=No] | | | | | | [Age in completed years] | | | | |
| **8.7** | Stroke (paralytic attack) | [1=Yes; 2=No] | | | | | | [Age in completed years] | | | | |
| **8.8** | Tuberculosis | [1=Yes; 2=No] | | | | | | [Age in completed years] | | | | |
| **8.9** | Asthma | [1=Yes; 2=No] | | | | | | [Age in completed years] | | | | |
| **8.10** | Peptic ulcer | [1=Yes; 2=No] | | | | | | [Age in completed years] | | | | |
| **8.11** | Thyroid problem | [1=Yes; 2=No] | | | | | | [Age in completed years] | | | | |
| **8.12** | Are you on any medication on a regular basis? | | | | | | | [1=Yes; 2=No] | | | | |
|  | If yes: | | | |  | | | | | | | |
| **8.13** | (a) Name of medicine | | | | (b) Reason for taking it (name of condition) | | | | | | | |
|  | __________________________ | | | | ________________________________ | | | | | | | |
|  | __________________________ | | | | ________________________________ | | | | | | | |
|  | __________________________ | | | | ________________________________ | | | | | | | |
|  | __________________________ | | | | ________________________________ | | | | | | | |
|  | __________________________ | | | | ________________________________ | | | | | | | |
|  | *Now some questions about your beliefs and feelings* | | | | | | | | | | | |
|  | **Your beliefs and feelings** | | | | | | |  | | | | |
| **9.1** | If you had a choice, where would you prefer to live? | | | | | | | [1=Village; 2=Town; 3=Small city;  4=Large city] | | | | |
| **9.2** | What is the primary reason for this preference? | | | | | | |  | | | | |
|  | [1=Economic/employment; 2=Availability of services (education/ leisure/healthcare/accessibility); 3=Family & other social ties; 4=Physical environment (housing/fresh air); 5=Way/speed of life; 6=Other, specify below] | | | | | | | | | | | |
| **9.3** | Other reason for place preference: ________________________________________ | | | | | | | | | | | |
| **9.4** | **In your opinion,** how much education should be given to girls these days? | | | | | | | | |  | | |
|  | [1=No education; 2=Literacy, but no formal education; 3=Up to primary school; 4=Secondary school; 5=Graduate; 6=Professional; 7=As much as she desires; 8=Depends; 9=Don’t know] | | | | | | | | | | | |
| **9.5** | **In your opinion,** should women wear western style dresses (e.g. jeans/tee-shirts)? | | | | | | | | | | |  |
|  | [1=No, never; 2=Wear at home but not outside; 3=Wear outside but not at home; 4=Wear at home & outside; 5=Don’t know] | | | | | | | | | | | |
| **9.6** | How often do you visit a place of worship outside home? | | | | | | | | |  | | |
|  | [1=Never; 2=Less than once a month; 3=More than once a month but less than once a week; 4=At least once a week] | | | | | | | | | | | |
| **9.7** | In an average week, how many days do you eat together as a family? | | | | | | | | | [0 – 7] | | |
| **9.8** | Do you think more and more people getting fat nowadays? | | | | | | | [1=Yes; 2=No; 3=Don’t know] | | | | |
| **9.9** | If yes, what are the important reasons for this change: | | | | | | | | | | | |
|  | (a) Less active lifestyle | | | | | | | [1=Yes; 2=No; 3=Don’t know] | | | | |
|  | (b) Eating more food in general | | | | | | | [1=Yes; 2=No; 3=Don’t know] | | | | |
|  | (c) Eating more sweet/oily/fatty food | | | | | | | [1=Yes; 2=No; 3=Don’t know] | | | | |
|  | (d) Food available is adulterated | | | | | | | [1=Yes; 2=No; 3=Don’t know] | | | | |
|  | (e) Eating outside of home more often | | | | | | | [1=Yes; 2=No; 3=Don’t know] | | | | |
|  | (f) Eating together as a family less often | | | | | | | [1=Yes; 2=No; 3=Don’t know] | | | | |
|  | (g) Any other reason not mentioned above | | | | _______________________________________ | | | | | | | |
| **9.10** | *About your feelings now, how often do you feel:* | | | | | | | | | | | |
|  |  | | | | | [1=Not at all; 2=Rarely; 3=Sometimes; 4=Often; 5=All the time] | | | | | | |
|  | (a) Lonely | | | | |  | | | | | | |
|  | (b) Missing friends and family | | | | |  | | | | | | |
|  | (c) Missing home comforts | | | | |  | | | | | | |
|  | (d) Insecure, stressed or anxious | | | | |  | | | | | | |
|  | (e) Frightened | | | | |  | | | | | | |
|  | (f) Tearful | | | | |  | | | | | | |
|  | (g) Sleepless | | | | |  | | | | | | |
|  | (h) Loss of appetite | | | | |  | | | | | | |
|  | (i) Loss of interest in usual activities | | | | |  | | | | | | |
|  | (j) Difficulty in concentrating | | | | |  | | | | | | |
|  | *Since you have moved from a village to a town/city, I would like to ask you some questions related to that.* | | | | | | | | | | | |
|  | **To be completed for rural-urban migrants only** | | | | | | |  | | | | |
| **10.1** | People can have many reasons for moving from village to live in a town or city. What was the most important reason in your case? | | | | | | | | |  | | |
|  | [1=Absolute lack of livelihood opportunity in rural area; 2= Better economic prospects/promotion in urban area; 3= Better availability of services (educational/ leisure/ healthcare / accessibility); 4= Social discrimination (caste); 5=Personal security (personal/ political reasons); 6= Social reasons (to be with family & friends/ marriage); 7=Natural disaster (floods/drought); 8=No clear reason/don’t know; 9=Any other reason not in the list (enter below)] | | | | | | | | | | | |
| **10.2** | Any other reason not in the list above: ______________________________________ | | | | | | | | | | | |
|  | *Thinking about when you first moved to the town/city:* | | | | | | | | | | | |
| **10.3** | How long did it take for you to feel at home/feel you belong/feel you are accepted in the WORKPLACE?( *Not to be filled for housewives).* | | | | | | | | |  | | |
|  | [1=Immediately; 2=Few weeks; 3=Few months; 4=More than a year; 5=Still don’t; 6=NA] | | | | | | | | | | | |
| **10.4** | How long did it take for you to feel at home/feel you belong/feel you are accepted in this TOWN/CITY? | | | | | | | | |  | | |
|  | [1=Immediately; 2=Few weeks; 3=Few months; 4=More than a year; 5=Still don’t] | | | | | | | | | | | |
| **10.5** | *Still thinking back to when you first moved to the town/city, did you feel:* | | | | | | | | | | | |
|  |  | | | | | [1=Not at all; 2=Rarely; 3=Sometimes; 4=Often; 5=All the time] | | | | | | |
|  | (a) Lonely | | | | |  | | | | | | |
|  | (b) Missing friends and family | | | | |  | | | | | | |
|  | (c) Missing home comforts | | | | |  | | | | | | |
|  | (d) Insecure, stressed or anxious | | | | |  | | | | | | |
|  | (e) Frightened | | | | |  | | | | | | |
|  | (f) Tearful | | | | |  | | | | | | |
|  | (g) Sleepless | | | | |  | | | | | | |
|  | (h) Loss of appetite | | | | |  | | | | | | |
|  | (i) Loss of interest in usual activities | | | | |  | | | | | | |
|  | (j) Difficulty in concentrating | | | | |  | | | | | | |
| **10.6** | If married, after how much time did your spouse move to live with you? | | | | | [In completed months; leave blank if  unmarried/spouse did not move] | | | | | | |
| **10.7** | Now thinking about your NATIVE PLACE (place of origin), what do you feel about the following: | | | | | | | | | | | |
|  |  | | | | | [1=Nothing at all; 2=Little; 3=Some; 4=Strong; 5=Very strong] | | | | | | |
|  | (a) Emotional attachment | | | | |  | | | | | | |
|  | (b) Respect from people there | | | | |  | | | | | | |
| **10.8** | During the preceding 2 years, how much time have you spent at your native place (place of origin) or other rural area? | | | | [1=None; 2=Less than 2 weeks; 3=Between 2-6  weeks; 3=More than 6 weeks] | | | | | | | |
| **10.9** | Do you send back regular (e.g. at least yearly) remittances to your family? | | | | [1=Yes; 2=No; 3=Not applicable] | | | | | | | |

|  | **Blood sampling** | |  | |
| --- | --- | --- | --- | --- |
| **11.1** | Any illness within the last week? | | [1=Yes; 2=No] | |
| **11.2** | If yes, specify what illness: _____________________________________________ | | | |
| **11.3** | Was this illness or some other reason responsible for reduction in food intake over the last week? | | [1=No reduction; 2=Minor  reduction; 3=Major reduction] | |
| **11.4** | Day of last meal | [1=Today; 2=Yesterday] | | |
| **11.5** | Time of last meal | :  [Hours: minutes; 24-hour clock] | | |
| **11.6** | Time blood taken | :  [Hours: minutes; 24-hour clock] | | |
|  | **Success in blood sampling** | | | |
|  |  | **(a) Volume**  **[1=No; 2=Partial; 3=Complete]** | | **(b) Clot formation**  **[1=Yes; 2=No**] |
| **11.7** | Red capped tube |  | |  |
| **11.8** | Purple capped tube 1 |  | |  |
| **11.9** | Grey capped tube |  | |  |
| **11.10** | Purple capped tube 2 |  | |  |
| **11.11** | (a) Any other comments about blood sample | [1=Yes; 2=No] | | |
|  | (b) If yes, specify |  | | |

|  | **Weight and height** | | |
| --- | --- | --- | --- |
| **12.1** | Weight | **.**  [kg] | |
| **12.2** | Weighing machine number |  | |
| **12.3** | Standing height | [mm] | |
| **12.4** | Stool height | [mm] | |
| **12.5** | Sitting height | [mm] | |
| **12.6** | Stadiometer number |  | |
|  | **Circumferences** | |  |
| **12.7** | Waist circumference 1 | [mm] | |
| **12.8** | Waist circumference 2 | [mm] | |
| **12.9** | Hip circumference 1 | [mm] | |
| **12.10** | Hip circumference 2 | [mm] | |
| **12.11** | Mid-arm circumference 1 | [mm] | |
| **12.12** | Mid-arm circumference 2 | [mm] | |
| **12.13** | Calf circumference 1 | [mm] | |
| **12.14** | Calf circumference 2 | [mm] | |
|  | **Skinfold measurements** | |  |
| **12.15** | Triceps skinfold 1 | **.**  [mm] | |
| **12.16** | Triceps skinfold 2 | **.**  [mm] | |
| **12.17** | Triceps skinfold 3 | **.**  [mm] | |
| **12.18** | Subscapular skinfold 1 | **.**  [mm] | |
| **12.19** | Subscapular skinfold 2 | **.**  [mm] | |
| **12.20** | Subscapular skinfold 3 | **.**  [mm] | |
| **12.21** | Calf skinfold 1 | **.**  [mm] | |
| **12.22** | Calf skinfold 2 | **.**  [mm] | |
| **12.23** | Calf skinfold 3 | **.**  [mm] | |
| **12.24** | Caliper number |  | |
|  | **General information: anthropometry measurements** | | |
| **12.25** | Researcher code |  | |
| **12.26** | Researcher initials |  | |
| **12.27** | Left sided measurements | [1=Yes; 2=No] | |
| **12.28** | If not, specify | __________________________________________ | |
| **12.29** | All measurements adequate | [1=Yes; 2=No] | |
| **12.30** | If not, specify | __________________________________________ | |

|  | Blood pressure | |
| --- | --- | --- |
| **13.1** | Researcher code |  |
| **13.2** | Researcher initials |  |
| **13.3** | Room temperature | **.**  [degree Celsius] |
| **13.4** | Systolic BP 1 | [mmHg] |
| **13.5** | Diastolic BP 1 | [mmHg] |
| **13.6** | Pulse rate 1 | [bpm] |
| **13.7** | Systolic BP 2 | [mmHg] |
| **13.8** | Diastolic BP 2 | [mmHg] |
| **13.9** | Pulse rate 2 | [bpm] |
| **13.10** | Cuff size used | [1=Small; 2=Medium; 3=Large] |
| **13.11** | BP apparatus number |  |
| **13.12** | Right arm measurements | [1=Yes; 2=No] |
| **13.13** | Measurements adequate | [1=Yes; 2=No] |
| **13.14** | If not, specify | __________________________________________ |

|  | Accelerometer |  |
| --- | --- | --- |
| **14.1** | Accelerometer number |  |
| **14.2** | Date of initiation | **___ ___ / ___ ___ / ___ ___** [DD/MM/YY] |
| **14.3** | Time of initiation | :  [Hours: minutes; 24-hour clock] |
| **14.4** | Date of termination | **___ ___ / ___ ___ / ___ ___** [DD/MM/YY] |
| **14.5** | Time of termination | Hours  Minutes |
| **14.6** | Comments | ________________________________________________ ________________________________________________ |

**SECTION** B**: Physical activity questionnaire (PAQ)**

|  | *Now I will ask you questions relating to your daily activity as this will help us to determine how active you are. Please answer these questions with respect to your activities over last ONE MONTH*. | | | | | | | | |
| --- | --- | --- | --- | --- | --- | --- | --- | --- | --- |
|  | **Work related activity** | |  | | | | | | |
| **1.1** | How many days in a week do you work? | | | | | | [In completed days] | | |
| **1.2** | On an average, how many hours per day do you spend at work? | | | | | | . [In completed half hours] | | |
| **1.3** | **Of the hours you spend at work, how many hours do you spend in (completed half hours):** | | | | | | | | |
|  | (a) **Standing:** Activities such as talk, lab work, supervise, mild cleaning, cattle grazing done standing. | (b) **Sitting:** Activities such as typing, computer work, cleaning grains, eating lunch, driving, ironing, done sitting etc | | (c) **Walking**: walking around, strolling | | | | (d) **On activities more strenuous than walking**: Fetch water/ fuel, fooder. weeding, chop wood, ploughing, pounding rice, walking with a load. | |
|  | . [hours] | . [hours] | | . [hours] | | | | . [hours] | |
| **1.4** | **If you spend any time at work on activities more strenuous than walking, please list the activities that you do most in terms of time:** | | | | | | | | |
|  | (a) | | | | | | | | |
|  | (b) | | | | | | | | |
|  | (c) | | | | | | | | |
|  | (d) | | | | | | | | |
| **1.5** | On an average, how many hours do you sleep in a day? | | | | | . [Completed half hours] | | | |
|  | **Apart from work, how do you spend your time (over the last month):**  Frequency options: [1=Daily; 2=Once a week; 3=2-4 times/week; 4=5-6 times/week; 5=2-3 times/month; 6=Once a month] | | | | | | | | |
| **2.1** | **Sports / games / exercise (for eg. walking, badminton, jogging, cricket………etc)** | | | | | | | | |
|  | (a) Name of activity | | | | (b) Duration | | | | (c) Frequency |
|  | _______________________________ | | | | [mts] | | | |  |
|  | _______________________________ | | | | [mts] | | | |  |
|  | _______________________________ | | | | [mts] | | | |  |
|  | _______________________________ | | | | [mts] | | | |  |
|  | _______________________________ | | | | [mts] | | | |  |
|  | _______________________________ | | | | [mts] | | | |  |
| **2.2** | **Hobbies involving manual labour (for eg. Carpentry, gardening ………….etc.)** | | | | | | | | |
|  | (a) Name of activity | | | | (b) Duration | | | | (c) Frequency |
|  | _______________________________ | | | | [mts] | | | |  |
|  | _______________________________ | | | | [mts] | | | |  |
|  | _______________________________ | | | | [mts] | | | |  |
|  | _______________________________ | | | | [mts] | | | |  |
|  | _______________________________ | | | | [mts] | | | |  |
|  | _______________________________ | | | | [mts] | | | |  |
| **2.3** | **Household activities (for eg. sweeping, collecting fuel/fodder/water, animal care, cooking, washing child care………. etc.)** | | | | | | | | |
|  | (a) Name of activity | | | | (b) Duration | | | | (c) Frequency |
|  | _______________________________ | | | | [mts] | | | |  |
|  | _______________________________ | | | | [mts] | | | |  |
|  | _______________________________ | | | | [mts] | | | |  |
|  | _______________________________ | | | | [mts] | | | |  |
|  | _______________________________ | | | | [mts] | | | |  |
|  | _______________________________ | | | | [mts] | | | |  |
|  | _______________________________ | | | | [mts] | | | |  |
|  | _______________________________ | | | | [mts] | | | |  |
|  | _______________________________ | | | | [mts] | | | |  |

|  | **Apart from work, how do you spend your time (over the last month):**  Frequency options: [1=Daily; 2=Once a week; 3=2-4 times/week; 4=5-6 times/week; 5=2-3 times/month; 6=Once a month] | | | |
| --- | --- | --- | --- | --- |
| **2.4** | **Sedentary activities for e.g. Reading, watching TV, prayer, carom, computer games, travelling……………. etc.)** | | | |
|  | (a) Name of activity | | (b) Duration | (c) Frequency |
|  | _______________________________ | | [mts] |  |
|  | _______________________________ | | [mts] |  |
|  | _______________________________ | | [mts] |  |
|  | _______________________________ | | [mts] |  |
|  | _______________________________ | | [mts] |  |
|  | _______________________________ | | [mts] |  |
|  | **Other activities** | | (a) Duration | (b) Frequency |
| **2.5** | Eating (breakfast, dinner) | | [mts] |  |
| **2.6** | Brushing, shaving & bathing | | [mts] |  |
| **2.7** | Dressing | | [mts] |  |
| **2.8** | Socialising (talking outside working hours) | | [mts] |  |
| **2.9** | Travelling to and fro from work | | [mts] |  |
| **2.10** | How do you travel to and fro from work? | _______________________________________ | | |

**SECTION C: Food Frequency Questionnaire (FFQ)**

| **INSTRUCTION TO SUBJECT:**  We are doing this study at a **national level** and there may be several food items in the list that you **may not have heard of** as they are eaten in other places. If you have not heard of an item please answer **“No”.** | | | | | | | | | | | | | | | | | | | |
| --- | --- | --- | --- | --- | --- | --- | --- | --- | --- | --- | --- | --- | --- | --- | --- | --- | --- | --- | --- |
|  | CEREALS | | **Portion**  **Size** | | | (a) Average consumption | | | | (b) PerDay^1^ | | | **(b) Per**  **Week^2^** | | | **(b) Per**  **Month^3^** | | | **(b) Per Year / Never^4^** |
| **1.1** | Tandoor roti, phulkas, wheat phulkas | | No | | |  | | | |  | | |  | | |  | | |  |
| **1.2** | Chapathis, parathas, naan | | No | | |  | | | |  | | |  | | |  | | |  |
| **1.3** | Stuffed parathas, franky | | No | | |  | | | |  | | |  | | |  | | |  |
| **1.4** | Rice roti | | No | | |  | | | |  | | |  | | |  | | |  |
| **1.5** | Ragi roti | | No | | |  | | | |  | | |  | | |  | | |  |
| **1.6** | Bajra, maize (makkai) roti | | No | | |  | | | |  | | |  | | |  | | |  |
| **1.7** | Jowar roti | | No | | |  | | | |  | | |  | | |  | | |  |
| **1.8** | Channa roti | | No | | |  | | | |  | | |  | | |  | | |  |
| **1.9** | Poori, bhatura | | No | | |  | | | |  | | |  | | |  | | |  |
| **1.10** | Plain rice | | Bowl | | |  | | | |  | | |  | | |  | | |  |
| **1.11** | Vegetable pulao/ veg biriyani | | Bowl | | |  | | | |  | | |  | | |  | | |  |
| **1.12** | Mutton, chicken pulao/biriyani | | Bowl | | |  | | | |  | | |  | | |  | | |  |
| **1.13** | Lime rice, puliogare, curd rice , tomato rice | | Bowl | | |  | | | |  | | |  | | |  | | |  |
| **1.14** | Bhagar | | Bowl | | |  | | | |  | | |  | | |  | | |  |
| **1.15** | Bisibelebhath | | Bowl | | |  | | | |  | | |  | | |  | | |  |
| **1.16** | Khichdi, khichri | | Bowl | | |  | | | |  | | |  | | |  | | |  |
| **1.17** | Pongal | | Bowl | | |  | | | |  | | |  | | |  | | |  |
| **1.18** | Upma | | Bowl | | |  | | | |  | | |  | | |  | | |  |
| **1.19** | Plain ragi ball | | No | | |  | | | |  | | |  | | |  | | |  |
| **1.20** | Ragi ball with rice | | No | | |  | | | |  | | |  | | |  | | |  |
| **1.21** | Idlis | | No | | |  | | | |  | | |  | | |  | | |  |
| **1.22** | Plain dosa, uthappam | | No | | |  | | | |  | | |  | | |  | | |  |
| **1.23** | Masala dosa | | No | | |  | | | |  | | |  | | |  | | |  |
| **1.24** | Pesarattu | | No | | |  | | | |  | | |  | | |  | | |  |
| **1.25** | Poha, Laia | | Bowl | | |  | | | |  | | |  | | |  | | |  |
| **1.26** | Avalakki, attakalu | | Bowl | | |  | | | |  | | |  | | |  | | |  |
| **1.27** | Dalia | | Bowl | | |  | | | |  | | |  | | |  | | |  |
|  | CEREALS (contd) | | **Portion**  **Size** | | | (a) Average consumption | | | | (b) PerDay^1^ | | | **(b) Per**  **Week^2^** | | | **(b) Per**  **Month^3^** | | | **(b) Per Year / Never^4^** |
| **1.28** | Rice, ragi porridge | | Bowl | | |  | | | |  | | |  | | |  | | |  |
| **1.29** | Vada, all types | | No | | |  | | | |  | | |  | | |  | | |  |
| **1.30** | Corn flakes, cereal flakes etc. | | Bowl | | |  | | | |  | | |  | | |  | | |  |
| **1.31** | Bread, Toast, Rolls, Buns | | No | | |  | | | |  | | |  | | |  | | |  |
| **1.32** | Pizza, Burger | | No | | |  | | | |  | | |  | | |  | | |  |
| **1.33** | Noodles, macaroni, pasta etc | | Bowl | | |  | | | |  | | |  | | |  | | |  |
|  | **LENTILS / DHALS / GRAVIES** | |  | | |  | | | |  | | |  | | |  | | |  |
| **2.1** | Plain tur dhal sambar / dhal | | Ladle | | |  | | | |  | | |  | | |  | | |  |
| **2.2** | Tur dhal sambar / dhal with vegetables | | Ladle | | |  | | | |  | | |  | | |  | | |  |
| **2.3** | Other dhal sambhar /dhals | | Ladle | | |  | | | |  | | |  | | |  | | |  |
| **2.4** | Channa, rajma, dry peas etc. curry | | Ladle | | |  | | | |  | | |  | | |  | | |  |
| **2.5** | Green leafy vegetable curry | | Ladle | | |  | | | |  | | |  | | |  | | |  |
| **2.6** | Paneer gravy | | Ladle | | |  | | | |  | | |  | | |  | | |  |
| **2.7** | Rasam, all types | | Ladle | | |  | | | |  | | |  | | |  | | |  |
| **2.8** | Kadhi | | Ladle | | |  | | | |  | | |  | | |  | | |  |
| **2.9** | Besan | | Ladle | | |  | | | |  | | |  | | |  | | |  |
| **2.10** | Mosaru huli | | Ladle | | |  | | | |  | | |  | | |  | | |  |
| **2.11** | Bassaaru, uppusaaru | | Ladle | | |  | | | |  | | |  | | |  | | |  |
| **2.12** | Mixed vegetable sagu | | Ladle | | |  | | | |  | | |  | | |  | | |  |
| **2.13** | Bengal gram sambar/curry | | Ladle | | |  | | | |  | | |  | | |  | | |  |
| **2.14** | Blackgram dhal curry | | Ladle | | |  | | | |  | | |  | | |  | | |  |
|  | **CHUTNEYS / SALAD / PAPAD** | |  | | |  | | | |  | | |  | | |  | | |  |
| **3.1** | Soups, all types (veg or non-veg) | | Bowl | | |  | | | |  | | |  | | |  | | |  |
| **3.2** | Fresh vegetable salad | | Tbsp | | |  | | | |  | | |  | | |  | | |  |
| **3.3** | Hesarebele salad | | Tbsp | | |  | | | |  | | |  | | |  | | |  |
| **3.4** | Vegetable Raitha | | Tbsp | | |  | | | |  | | |  | | |  | | |  |
| **3.5** | Mango, lime pickle etc. | | Tsp | | |  | | | |  | | |  | | |  | | |  |
| **3.6** | Papad | | No | | |  | | | |  | | |  | | |  | | |  |
| **3.7** | Kachri | | Bowl | | |  | | | |  | | |  | | |  | | |  |
| **3.8** | Sandige, vathal | | No | | |  | | | |  | | |  | | |  | | |  |
| **3.9** | Coconut chutney | | Tbsp | | |  | | | |  | | |  | | |  | | |  |
|  | **CHUTNEYS / SALAD / PAPAD**  **(contd)** | | **Portion**  **Size** | | | (a) Average consumption | | | | (b) PerDay^1^ | | | **(b) Per**  **Week^2^** | | | **(b) Per**  **Month^3^** | | | **(b) Per Year / Never^4^** |
| **3.10** | Groundnut chutney | | Tbsp | | |  | | | |  | | |  | | |  | | |  |
| **3.11** | Tomato chutney | | Tbsp | | |  | | | |  | | |  | | |  | | |  |
| **3.12** | Chilli chutney | | Tbsp | | |  | | | |  | | |  | | |  | | |  |
| **3.13** | Tamarind chutney | | Tbsp | | |  | | | |  | | |  | | |  | | |  |
| **3.14** | Mango chutney | | Tbsp | | |  | | | |  | | |  | | |  | | |  |
| **3.15** | Brinjal, ridgegourd,other vegetable chutney | | Tbsp | | |  | | | |  | | |  | | |  | | |  |
| **3.16** | Gogu chutney | | Tbsp | | |  | | | |  | | |  | | |  | | |  |
| **3.17** | Urad dhal chutney | | Tbsp | | |  | | | |  | | |  | | |  | | |  |
| **3.18** | Varhadi Thecha | | Tbsp | | |  | | | |  | | |  | | |  | | |  |
| **3.19** | Chutney powder | | Tbsp | | |  | | | |  | | |  | | |  | | |  |
|  | **NON – VEGETARIAN** | |  | | |  | | | |  | | |  | | |  | | |  |
| **4.1** | Chicken curry | | Bowl | | |  | | | |  | | |  | | |  | | |  |
| **4.2** | Chicken fry/grilled | | No | | |  | | | |  | | |  | | |  | | |  |
| **4.3** | Mutton/ pork/beef curry | | Bowl | | |  | | | |  | | |  | | |  | | |  |
| **4.4** | Mutton / beef/ pork / fry | | No. | | |  | | | |  | | |  | | |  | | |  |
| **4.5** | Fish curry | | Bowl | | |  | | | |  | | |  | | |  | | |  |
| **4.6** | Fish fry | | No | | |  | | | |  | | |  | | |  | | |  |
| **4.7** | Organ meats (Liver, brain, kidney etc.) | | Tbsp | | |  | | | |  | | |  | | |  | | |  |
| **4.8** | Prawn, crab, shell fish etc. | | Bowl | |  | | | |  | | |  | | |  | | |  | |
| **4.9** | Egg (boiled, poached, omelettes) | | No | |  | | | |  | | |  | | |  | | |  | |
| **4.10** | Mutton, chicken etc Kebabs | | No | |  | | | |  | | |  | | |  | | |  | |
| **4.11** | Ham, salami, bacon etc. | | Slices | |  | | | |  | | |  | | |  | | |  | |
| **4.12** | Pigeon | | Bowl | |  | | | |  | | |  | | |  | | |  | |
| **4.13** | Other poultry (lave, titar, bater etc) | | Bowl | |  | | | |  | | |  | | |  | | |  | |
| **4.14** | Rabbit | | Bowl | |  | | | |  | | |  | | |  | | |  | |
|  | **MILK & BEVERAGES** | |  | |  | | | |  | | |  | | |  | | |  | |
| **5.1** | Tea | | Glass | |  | | | |  | | |  | | |  | | |  | |
| **5.2** | Coffee | | Glass | |  | | | |  | | |  | | |  | | |  | |
| **5.3** | Plain milk | | Glass | |  | | | |  | | |  | | |  | | |  | |
| **5.4** | Flavored milk (horlicks, bournvita etc) | | Glass | |  | | | |  | | |  | | |  | | |  | |
| **5.5** | Curd, yoghurt | | Bowl | |  | | | |  | | |  | | |  | | |  | |
|  | **MILK & BEVERAGES (contd)** | | **Portion**  **Size** | | (a) Average consumption | | | | (b) PerDay^1^ | | | **(b) Per**  **Week^2^** | | | **(b) Per**  **Month^3^** | | | **(b) Per Year / Never^4^** | |
| **5.6** | Buttermilk/Lassi | | Glass | |  | | | |  | | |  | | |  | | |  | |
| **5.7** | Fresh fruit juice(lime, orange etc) | | Glass | |  | | | |  | | |  | | |  | | |  | |
| **5.8** | Fanta, pepsi, coca cola etc. | | 250ml bottle | |  | | | |  | | |  | | |  | | |  | |
| **5.9** | Beer | | Glass | |  | | | |  | | |  | | |  | | |  | |
| **5.10** | Wine | | Glass | |  | | | |  | | |  | | |  | | |  | |
| **5.11** | Spirits (whiskey, gin, rum) | | 30ml peg | |  | | | |  | | |  | | |  | | |  | |
| **5.12** | Local arrack/toddy | | Glass | |  | | | |  | | |  | | |  | | |  | |
| **5.13** | Aam ka panna | | Glass | |  | | | |  | | |  | | |  | | |  | |
|  | **MISCELLANEOUS** | |  | |  | | | |  | | |  | | |  | | |  | |
| **6.1** | Butter/ cream | | Tsp | |  | | | |  | | |  | | |  | | |  | |
| **6.2** | Ghee | | Tsp | |  | | | |  | | |  | | |  | | |  | |
| **6.3** | Jam | | Tsp | |  | | | |  | | |  | | |  | | |  | |
| **6.4** | Sugar | | Tsp | |  | | | |  | | |  | | |  | | |  | |
| **6.5** | Honey | | Tsp | |  | | | |  | | |  | | |  | | |  | |
| **6.6** | Jaggery | | Tsp | |  | | | |  | | |  | | |  | | |  | |
| **6.7** | Cheese | | Cube | |  | | | |  | | |  | | |  | | |  | |
| **6.8** | Ketchup, tomato sauce | | Tbsp | |  | | | |  | | |  | | |  | | |  | |
|  | **SNACKS/ SWEETS/DESSERTS** | |  | |  | | | |  | | |  | | |  | | |  | |
| **7.1** | Mixture, namkeen, chiwda, khara boondi, dalmoth | | Tbsp | |  | | | |  | | |  | | |  | | |  | |
| **7.2** | Nuts (grounduts, cashewnuts etc.) | | Tbsp | |  | | | |  | | |  | | |  | | |  | |
| **7.3** | Chips, French fries | | Bowl | |  | | | |  | | |  | | |  | | |  | |
| **7.4** | Samosa,bajji ,bonda, cutlet, patties | | No | |  | | | |  | | |  | | |  | | |  | |
| **7.5** | Veg & non-veg puff | | No | |  | | | |  | | |  | | |  | | |  | |
| **7.6** | Biscuits (salted) | | No | |  | | | |  | | |  | | |  | | |  | |
| **7.7** | Biscuits (sweet, creamed, etc) | | No | |  | | | |  | | |  | | |  | | |  | |
| **7.8** | Bhel puri, masala puri, other chaats | | Bowl | |  | | | |  | | |  | | |  | | |  | |
| **7.9** | Murukku , chakli, sakinalu | | No | |  | | | |  | | |  | | |  | | |  | |
| **7.10** | Dhokla | | No | |  | | | |  | | |  | | |  | | |  | |
| **7.11** | Pav bhaji | | No | |  | | | |  | | |  | | |  | | |  | |
| **7.12** | Cakes or sweet pastries | | No | |  | | | |  | | |  | | |  | | |  | |
| **7.13** | Payasam, kheer | | Bowl | |  | | | |  | | |  | | |  | | |  | |
|  | **SNACKS/ SWEETS/DESSERTS**  **(contd)** | | **Portion**  **Size** | | (a) Average consumption | | | | (b) PerDay^1^ | | | **(b) Per**  **Week^2^** | | | **(b) Per**  **Month^3^** | | | **(b) Per Year / Never^4^** | |
| **7.14** | Custard, puddings | | Bowl | |  | | | |  | | |  | | |  | | |  | |
| **7.15** | Ice cream | | Bowl | |  | | | |  | | |  | | |  | | |  | |
| **7.16** | Jamoon, Jilebi, Jangir etc. | | No | |  | | | |  | | |  | | |  | | |  | |
| **7.17** | Mysore pak, ladoo, barfis | | No | |  | | | |  | | |  | | |  | | |  | |
| **7.18** | Indian milk sweet (peda, rasgulla etc. | | No | |  | | | |  | | |  | | |  | | |  | |
| **7.19** | All Halwas | | Tbsp | |  | | | |  | | |  | | |  | | |  | |
| **7.20** | Puran poli, obattu/holige | | No | |  | | | |  | | |  | | |  | | |  | |
| **7.21** | Shakarpara, balushahi, badusha | | No | |  | | | |  | | |  | | |  | | |  | |
| **7.22** | Kesari bhath | | Tbsp | |  | | | |  | | |  | | |  | | |  | |
| **7.23** | Kajjaya, karjikayi | | No | |  | | | |  | | |  | | |  | | |  | |
| **7.24** | Kadubu | | No | |  | | | |  | | |  | | |  | | |  | |
| **7.25** | Baksham, arisalu, poornalu | | No | |  | | | |  | | |  | | |  | | |  | |
| **7.26** | Sweet pongal | | Bowl | |  | | | |  | | |  | | |  | | |  | |
| **7.27** | Sonpapdi, kaju katli | | No | |  | | | |  | | |  | | |  | | |  | |
| **7.28** | Gujiya | | No | |  | | | |  | | |  | | |  | | |  | |
| **7.29** | Shirkurama | | Bowl | |  | | | |  | | |  | | |  | | |  | |
| **7.30** | Shrikand | | Bowl | |  | | | |  | | |  | | |  | | |  | |
| **7.31** | Dairy milk, 5 star, kitkat etc. | | Small Bar | |  | | | |  | | |  | | |  | | |  | |
|  | | | | | | | | | | | | | | | | | | | |
|  | **FRUITS** | **Portion size** | | (a) Average consumption | | | (b) PerDay^1^ | **(b) Per**  **Week^2^** | | | **(b) Per**  **Month^3^** | | | **(b) Per Year/ Never^4^** | | | (c) Seasonal **(cross if seasonal)** | | |
| **8.1** | Banana | No | |  | | |  |  | | |  | | |  | | |  | | |
| **8.2** | Apple | No | |  | | |  |  | | |  | | |  | | |  | | |
| **8.3** | Orange | No | |  | | |  |  | | |  | | |  | | |  | | |
| **8.4** | Sweet lime | No | |  | | |  |  | | |  | | |  | | |  | | |
| **8.5** | Mango | No | |  | | |  |  | | |  | | |  | | |  | | |
| **8.6** | Guava (amrood) | No | |  | | |  |  | | |  | | |  | | |  | | |
| **8.7** | Grapes (angoor) | Bowl | |  | | |  |  | | |  | | |  | | |  | | |
| **8.8** | Pineapple | Slice | |  | | |  |  | | |  | | |  | | |  | | |
| **8.9** | Papaya ( papita) | Slice | |  | | |  |  | | |  | | |  | | |  | | |
| **8.10** | Pomegranate ( anar) | No | |  | | |  |  | | |  | | |  | | |  | | |
|  | **FRUITS (contd)** | **Portion size** | | (a) Average consumption | | | (b) PerDay^1^ | **(b) Per**  **Week^2^** | | | **(b) Per**  **Month^3^** | | | **(b) Per Year/ Never^4^** | | | (c) Seasonal **(cross if seasonal)** | | |
| **8.11** | Sapota ( Chikoo) | No | |  | | |  |  | | |  | | |  | | |  | | |
| **8.12** | Watermelon ( tarbooj) | Bowl | |  | | |  |  | | |  | | |  | | |  | | |
| **8.13** | Musk melon ( kharbooj) | Bowl | |  | | |  |  | | |  | | |  | | |  | | |
| **8.14** | Jackfruit | No | |  | | |  |  | | |  | | |  | | |  | | |
| **8.15** | Custard apple | No | |  | | |  |  | | |  | | |  | | |  | | |
| **8.16** | Plums | No | |  | | |  |  | | |  | | |  | | |  | | |
| **8.17** | Zizyphus (ber) | No | |  | | |  |  | | |  | | |  | | |  | | |
| **8.18** | Sugarcane (ganaa) | Pieces | |  | | |  |  | | |  | | |  | | |  | | |
| **8.19** | Litchis | No | |  | | |  |  | | |  | | |  | | |  | | |
| **8.20** | Pears | No | |  | | |  |  | | |  | | |  | | |  | | |
| **8.21** | Peaches | No | |  | | |  |  | | |  | | |  | | |  | | |
| **8.22** | Kiwi | No | |  | | |  |  | | |  | | |  | | |  | | |
| **8.23** | Jamoon | No | |  | | |  |  | | |  | | |  | | |  | | |
| **8.24** | Palmyra | No | |  | | |  |  | | |  | | |  | | |  | | |
| **8.25** | Amla | No | |  | | |  |  | | |  | | |  | | |  | | |
| **8.26** | Fruit salad | Bowl | |  | | |  |  | | |  | | |  | | |  | | |
| **8.27** | Dried fruits (dates, figs, raisins etc) | No | |  | | |  |  | | |  | | |  | | |  | | |
|  | **VEGETABLES** |  | |  | | |  |  | | |  | | |  | | |  | | |
| **9.1** | Palak, methi, other leafy vegetables | Tbsp | |  | | |  |  | | |  | | |  | | |  | | |
| **9.2** | Potato, sweet potato | Tbsp | |  | | |  |  | | |  | | |  | | |  | | |
| **9.3** | Carrot | Tbsp | |  | | |  |  | | |  | | |  | | |  | | |
| **9.4** | Beetroot/ radish/ knol-khol | Tbsp | |  | | |  |  | | |  | | |  | | |  | | |
| **9.5** | Cabbage | Tbsp | |  | | |  |  | | |  | | |  | | |  | | |
| **9.6** | Beans, cluster beans | Tbsp | |  | | |  |  | | |  | | |  | | |  | | |
| **9.7** | Ladies finger | Tbsp | |  | | |  |  | | |  | | |  | | |  | | |
| **9.8** | Cauliflower | Tbsp | |  | | |  |  | | |  | | |  | | |  | | |
| **9.9** | Bottlegourd(lauki),ashgourd,Ridgegourd(turai), snakegourds, etc. | Tbsp | |  | | |  |  | | |  | | |  | | |  | | |
| **9.10** | Brinjal | Tbsp | |  | | |  |  | | |  | | |  | | |  | | |
| **9.11** | Mushrooms | Tbsp | |  | | |  |  | | |  | | |  | | |  | | |
| **9.12** | Fresh peas | Tbsp | |  | | |  |  | | |  | | |  | | |  | | |
| **9.13** | Pumpkin | Tbsp | |  | | |  |  | | |  | | |  | | |  | | |
|  | **VEGETABLES (Contd)** | **Portion size** | | (a) Average consumption | | | (b) PerDay^1^ | **(b) Per**  **Week^2^** | | | **(b) Per**  **Month^3^** | | | **(b) Per Year/ Never^4^** | | | (c) Seasonal **(cross if seasonal)** | | |
| **9.14** | Parwal, kovai | Tbsp | |  | | |  |  | | |  | | |  | | |  | | |
| **9.15** | Capsicum or green pepper | Tbsp | |  | | |  |  | | |  | | |  | | |  | | |
| **9.16** | Drumstick | Pieces | |  | | |  |  | | |  | | |  | | |  | | |
| **9.17** | Raw plantain | Tbsp | |  | | |  |  | | |  | | |  | | |  | | |
| **9.18** | Colacasia (arvi) | Tbsp | |  | | |  |  | | |  | | |  | | |  | | |
| **9.19** | Jackfruit tender | Tbsp | |  | | |  |  | | |  | | |  | | |  | | |
| **9.20** | Dhemsa | Tbsp | |  | | |  |  | | |  | | |  | | |  | | |
| **9.21** | Karela | Tbsp | |  | | |  |  | | |  | | |  | | |  | | |
| **9.22** | Tinda | Tbsp | |  | | |  |  | | |  | | |  | | |  | | |
| **9.23** | Lotus stem | Tbsp | |  | | |  |  | | |  | | |  | | |  | | |
| **9.24** | Chow chow marrow | Tbsp | |  | | |  |  | | |  | | |  | | |  | | |
| **9.25** | Yam | Tbsp | |  | | |  |  | | |  | | |  | | |  | | |

| **10.1** | How many liters of these oils / fats does your family consume in a month? (Kg /month) | | | |
| --- | --- | --- | --- | --- |
|  | (a) Sunflower oil | | . | |
|  | (b) Groundnut oil | | .. | |
|  | (c) Coconut oil | | . | |
|  | (d) Palm Oil | | . | |
|  | (e) Mustard oil | | . | |
|  | (f) Dalda / vanaspathi | | . | |
|  | (g) Butter | | . | |
|  | (h) Ghee | | . | |
|  | (i) Olive oil | | . | |
|  | (j) Corn oil | | . | |
|  | (k)Rice bran oil | | . | |
|  | (l) Soya bean oil | | . | |
|  | (m) Others | | . | |
| **10.2** | If others, please specify | |  | |
| **10.3** | Do you routinely remove fat / skin from meat before cooking? | | [1=Yes; 2=No] | |
| **10.4** | How many coconuts do you use for cooking in a month? (No / month) | |  | |
| **10.5** | Do you add any of the following as a thickening agent for your curries or vegetables at least 2 times a week? | |  | |
|  | (a) Coconut | | [1=Yes; 2=No] | |
|  | (b) Groundnuts | | [1=Yes; 2=No] | |
|  | (c) Roasted Bengal gram | | [1=Yes; 2=No] | |
| **10.6** | What type of milk do you regularly consume? | |  | |
|  | [1=Whole milk, 2=Skimmed Milk, 3=Toned milk, 4=Skimmed milk powder] | | | |
| **10.7** | Do you consume any vitamin or mineral supplement at least once a week?  [1=Yes; 2=No] | | | |
| **10.8** | If Yes, | | | |
|  | (a) **Brand name / Type** | (b) **Dosage(mg)** | | (c) **No. / week** |
|  |  |  | |  |
|  |  |  | |  |
|  |  |  | |  |
|  |  |  | |  |
| **10.9** | Are you on any special diet? | [1=Yes; 2=No] | | |
| **10.10** | If yes, | | | |
|  | (a) Diabetic diet | [1=Yes; 2=No] | | |
|  | (b) Low fat diet | [1=Yes; 2=No] | | |
|  | (c) High fiber diet | [1=Yes; 2=No] | | |
|  | (d) Low salt diet | [1=Yes; 2=No] | | |
|  | (e) Weight reducing diet | [1=Yes; 2=No] | | |
|  | (f) Others | [1=Yes; 2=No] | | |
| **10.11** | If others, please specify | _____________________________ | | |
| **10.12** | Since how many years are you on this special diet? | [years] | | |

**SECTION D: Consent form**

**Study Title:** Rural- urban migration: effects on obesity and diabetes in Indians.

**Participant:**

Shri/Smt/Kum (First & Last Name)

Address (Lane, Town, State, Pin Code)

- I am free to participate or not to participate in this study.
- I have been given the opportunity to ask questions and reply was given for all the questions to my satisfaction.
- I have been informed by the investigators about the process including the nature, objective and known and likely inconveniences related to this study and I have understood them.
- My medical data are strictly confidential and I only authorise the persons, involved in the research, identified by the sponsor or health authorities to consult about the same.
- By signing this form, I give my free and informed consent to take part in this study as outlined in the information sheet and this consent form. Specifically, I agree to being interviewed, examined and having blood drawn. I also agree to my information, including results of blood tests, to be used in research.
- I give permission for any blood that is left over after the tests to be stored and used for further laboratory tests for medical research
- I understand that future research using the sample I give may include genetic research aimed at understanding genetic influences on diseases but the results of these investigations are unlikely to have any implications for you personally
- I have been given a copy of the information sheet and consent form to keep. By signing this form I have not given up my legal rights.

Printed name of the Participant______________________________________________

Signature of the Participant___________________________________Date________________________

Printed name of the Investigator_____________________________________________

Signature of the Investigator__________________________________Date________________________
