# Supplementary material for: Relative contribution of diet and physical activity to increased adiposity among rural to urban migrants in India: A cross-sectional study
Source: PLoS Med. 2020 Aug 7;17(8):e1003234. doi: 10.1371/journal.pmed.1003234 (PMC7413404; doi:10.1371/journal.pmed.1003234)
Supplement: S1 Fig — (DOC) [file pmed.1003234.s003.doc]

**S1 Fig.** Recruitment flow chart, Indian Migration Study, 2005-2007

7,102 agreed in principle to participate with their sibling.

15,596 subjects (workers/ spouses) identified & contacted

13,695 subjects completed assessment of eligibility.

7,594 subjects were eligible.

1901 subjects did not complete assessments.

6101 were not eligible. Did not have a rural sib or were not randomly chosen for the urban non-migrant sample.

492 did not want to participate.

7,067 individuals (worker, spouse or sibling) participated:

38 Urban-rural migrant workers & their sibs

519 Rural workers & their sibs

4,223 Rural-urban migrant workers & their sibs

2,287 Urban workers & their sibs

3,537 workers or spouses (and 3530 siblings) participated by the end of study.

(3,525 sib-pairs; unpaired - 12 workers, 5 siblings)

2108 urban migrants with a rural sibling of either sex

1232 urban migrants with a rural sibling of the same sex (N=2482)

3565 had not participated by the end of study.
