# Supplementary material for: Relative contribution of diet and physical activity to increased adiposity among rural to urban migrants in India: A cross-sectional study
Source: PLoS Med. 2020 Aug 7;17(8):e1003234. doi: 10.1371/journal.pmed.1003234 (PMC7413404; doi:10.1371/journal.pmed.1003234)
Supplement: S1 Table — (DOC) [file pmed.1003234.s004.doc]

S1 Table. Participant characteristics by response, Indian Migration Study, 2005-2007

| **Characteristics** | **Responders** | **Non-responders** | **No consent** |
| --- | --- | --- | --- |
| N | 3537 | 3565 | 492 |
| Male, N (%) | 1,800 (50.9) | 2,057 (57.7) | 279 (56.7) |
| Age, mean (SD) | 41.7 (9.2) | 41.9 (9.6) | 46.2 (7.9) |
| Hindu religion, N (%) | 3,243 (91.7) | 3,345 (93.8) | 446 (90.7) |
| Married, N (%) | 3,436 (97.1) | 3,439 (96.5) | 478 (97.1) |
| Self-reported high blood pressure, heart disease or stroke, N (%) | 684 (19.3) | 526 (14.8) | 104 (21.1) |
| Currently smoke/chew tobacco, N (%) | 646 (18.3) | 723 (20.3) | 80 (16.3) |
| Distance in hours from factory to place of origin, Mean (SD) (Q1; Q3) | 6.5 (7.0)  (2; 9) | 6.8 (8.0)  (1; 9) | 8.2 (9.6)  (2; 12) |
| Migrant, N (%) | 2,112 (59.7) | 2,165 (60.7) | 372 (75.6) |
