# Supplementary material for: Relative contribution of diet and physical activity to increased adiposity among rural to urban migrants in India: A cross-sectional study
Source: PLoS Med. 2020 Aug 7;17(8):e1003234. doi: 10.1371/journal.pmed.1003234 (PMC7413404; doi:10.1371/journal.pmed.1003234)
Supplement: S2 Table — (DOCX) [file pmed.1003234.s005.docx]

**S2 Table.** Multivariable model for association of difference in energy intake and energy expenditure on physical activity on the difference in % body fat between urban and rural siblings, adjusted for difference in Standard of Living Index, in the Indian Migration Study, 2005-2007

| **Variable** | | **β** | **95% CI** | **p-value** |
| --- | --- | --- | --- | --- |
| Energy intake (calories/day) | | 0.0001 | (-0.000, 0.001) | 0.109 |
| Physical activity energy expenditure (kj/kg/day) | | -0.016 | (-0.031, -0.001) | 0.042 |
| Age (years) | | 0.216 | (0.176, 0.256) | <0.001 |
| Sex (female) | | -1.458 | (-2.229, -0.686) | <0.001 |
| Years lived in urban area (per year) | | 0.021 | (-0.024, 0.066) | 0.355 |
| Factory site | Lucknow | Ref. | - | - |
|  | Nagpur | -0.183 | (-1.424, 1.059) | 0.773 |
|  | Hyderabad | -0.751 | (-1.688, 0.187) | 0.116 |
|  | Bangalore | -0.757 | (-1.868, 0.355) | 0.182 |
| Standard of Living Index | | 0.262 | (0.208, 0.316) | <0.001 |

N=2216 (1108 pairs). Participants with complete data only

β is beta-coefficient, CI is confidence intervals

Variables in the table are mutually adjusted for each other and rural sibling used as the reference.
