# Supplementary material for: Relative contribution of diet and physical activity to increased adiposity among rural to urban migrants in India: A cross-sectional study
Source: PLoS Med. 2020 Aug 7;17(8):e1003234. doi: 10.1371/journal.pmed.1003234 (PMC7413404; doi:10.1371/journal.pmed.1003234)
Supplement: S3 Table — (DOCX) [file pmed.1003234.s006.docx]

**S3 Table.** Multivariable model for association of difference in selected diet and physical activity behaviours on the difference in % body fat between urban and rural siblings, adjusted for difference in Standard of Living Index, in the Indian Migration Study, 2005-2007

| **Variable** | | **β** | **95% CI** | **p-value** |
| --- | --- | --- | --- | --- |
| Cereal & legume intake (grams/day) | | 0.000 | (-0.002, 0.002) | 0.908 |
| Meat, fish & poultry intake (grams/day) | | 0.004 | (-0.006, 0.014) | 0.418 |
| Dairy intake (grams/day) | | 0.001 | (-0.001, 0.002) | 0.459 |
| Fruit & vegetable intake (grams/day) | | -0.001 | (-0.003, 0.001) | 0.284 |
| Sugary food and sweets intake (grams/day) | | 0.000 | (-0.019, 0.018) | 0.965 |
| Fats & oils intake (grams/day) | | 0.015 | (-0.005, 0.035) | 0.150 |
| Time spent sedentary (min/day) | | 0.000 | (-0.002, 0.002) | 0.765 |
| Time spent in moderate or vigorous activity (min/day) | | -0.002 | (-0.005, 0.001) | 0.190 |
| Time spent watching television (min/day) | | 0.003 | (-0.002, 0.007) | 0.219 |
| Age (year) | | 0.217 | (0.178, 0.257) | <0.001 |
| Sex (female) | | -1.514 | (-2.291, -0.737) | <0.001 |
| Years lived in urban area (per year) | | 0.021 | (-0.025, 0.066) | 0.372 |
| Factory site | Lucknow | Ref. | - | - |
|  | Nagpur | -0.670 | (-2.084, 0.744) | 0.353 |
|  | Hyderabad | -1.103 | (-2.169, -0.037) | 0.043 |
|  | Bangalore | -1.033 | (-2.253, 0.187) | 0.097 |
| Standard of Living Index | | 0.236 | (0.181, 0.292) | <0.001 |

N=2282 (1141 pairs). Participants with complete data only

β is beta-coefficient, CI is confidence intervals

Variables in the table are mutually adjusted for each other and rural sibling used as the reference
