# Supplementary material for: Relative contribution of diet and physical activity to increased adiposity among rural to urban migrants in India: A cross-sectional study
Source: PLoS Med. 2020 Aug 7;17(8):e1003234. doi: 10.1371/journal.pmed.1003234 (PMC7413404; doi:10.1371/journal.pmed.1003234)
Supplement: S4 Table — (DOCX) [file pmed.1003234.s007.docx]

**S4 Table.** Multivariable model for association of difference in energy intake and energy expenditure on physical activity on the difference in % body fat between urban and rural siblings, stratified by the sex of the sibling pair, in the Indian Migration Study, 2005-2007

| **Variable** | | **Males (n=759 pairs)** | | | **Females (n=349 pairs)** | | |
| --- | --- | --- | --- | --- | --- | --- | --- |
|  |  | **β** | **95% CI** | **p-value** | **β** | **95% CI** | **p-value** |
| Energy intake (calories/day) | | 0.001 | (0.0001, 0.001) | 0.005 | 0.0004 | (-0.0004, 0.001) | 0.321 |
| Physical activity energy expenditure (kj/kg/day) | | -0.038 | (-0.057, -0.019) | <0.001 | -0.018 | (-0.045, 0.009) | 0.189 |
| Age (years) | | 0.216 | (0.167, 0.266) | <0.001 | 0.214 | (0.139, 0.290) | <0.001 |
| Years lived in urban area (per year) | | 0.039 | (-0.019, 0.098) | 0.190 | 0.056 | (-0.021, 0.133) | 0.153 |
| Factory site | Lucknow | Ref. | - | - | Ref. | - | - |
|  | Nagpur | -0.825 | (-2.32, 0.671) | 0.280 | -4.45 | (-6.84, -2.07) | <0.001 |
|  | Hyderabad | 0.149 | (-1.07, 1.36) | 0.809 | -2.21 | (-3.92, -0.504) | 0.011 |
|  | Bangalore | -0.282 | (-1.68, 1.12) | 0.692 | -3.54 | (-5.63, -1.46) | 0.001 |

N=2216 (1108 pairs). Participants with complete data only

β is beta-coefficient, CI is confidence intervals

Variables in the table are mutually adjusted for each other and rural sibling used as the reference

There were no significant interactions by sex (p>0.1).
