# Supplementary material for: Relative contribution of diet and physical activity to increased adiposity among rural to urban migrants in India: A cross-sectional study
Source: PLoS Med. 2020 Aug 7;17(8):e1003234. doi: 10.1371/journal.pmed.1003234 (PMC7413404; doi:10.1371/journal.pmed.1003234)
Supplement: S5 Table — (DOCX) [file pmed.1003234.s008.docx]

**S5 Table.** Multivariable model for association of difference in selected diet and physical activity behaviours on the difference in % body fat between urban and rural siblings, stratified by the sex of the sibling pair, in the Indian Migration Study 2005-2007

| **Variable** | | **Males (n=794 pairs)** | | | **Females (n=347 pairs)** | | |
| --- | --- | --- | --- | --- | --- | --- | --- |
|  |  | **β** | **95% CI** | **p-value** | **β** | **95% CI** | **p-value** |
| Cereal & legume intake (grams/day) | | 0.000 | (-0.002,0.002) | 0.988 | -0.002 | (-0.006,0.003) | 0.451 |
| Meat, fish & poultry intake (grams/day) | | 0.003 | (-0.009,0.015) | 0.661 | 0.02 | (-0.001,0.042) | 0.058 |
| Dairy intake (grams/day) | | 0.002 | (-0.001,0.004) | 0.146 | 0.001 | (-0.003,0.005) | 0.57 |
| Fruit & vegetable intake (grams/day) | | 0.000 | (-0.002,0.002) | 0.977 | -0.002 | (-0.005,0.002) | 0.354 |
| Sugary food and sweets intake (grams/day) | | -0.001 | (-0.023,0.021) | 0.938 | -0.007 | (-0.042,0.029) | 0.712 |
| Fats & oils intake (grams/day) | | 0.011 | (-0.014,0.036) | 0.394 | 0.038 | (-0.001,0.078) | 0.056 |
| Time spent sedentary (min/day) | | 0.001 | (-0.002,0.004) | 0.514 | 0.000 | (-0.004,0.003) | 0.909 |
| Time spent in moderate or vigorous activity (min/day) | | -0.004 | (-0.007,-0.000) | 0.045 | -0.002 | (-0.009,0.004) | 0.425 |
| Time spent watching television (min/day) | | 0.008 | (0.002,0.014) | 0.008 | 0.004 | (-0.003,0.010) | 0.274 |
| Age (year) | | 0.214 | (0.166,0.263) | <0.001 | 0.222 | (0.147,0.298) | <0.001 |
| Years lived in urban area (per year) | | 0.037 | (-0.021,0.095) | 0.212 | 0.044 | (-0.034,0.121) | 0.268 |
| Factory site | Lucknow | Ref. | - | - | Ref. | - | - |
|  | Nagpur | -1.055 | (-2.754,0.643) | 0.223 | -5.857 | (-8.506,-3.208) | <0.001 |
|  | Hyderabad | -0.297 | (-1.681,1.087) | 0.674 | -3.112 | (-5.012,-1.211) | 0.001 |
|  | Bangalore | -0.743 | (-2.280,0.793) | 0.343 | -3.751 | (-5.973,-1.530) | 0.001 |

N=2282 (1141 pairs). Participants with complete data only

β is beta-coefficient, CI is confidence intervals

Variables in the table are mutually adjusted for each other and rural sibling used as the reference

There were no significant interactions by sex (p>0.1).
