# Supplementary material for: Relative contribution of diet and physical activity to increased adiposity among rural to urban migrants in India: A cross-sectional study
Source: PLoS Med. 2020 Aug 7;17(8):e1003234. doi: 10.1371/journal.pmed.1003234 (PMC7413404; doi:10.1371/journal.pmed.1003234)
Supplement: Attachment — Submitted filename: Response to reviewers_Kinra et al.docx [file pmed.1003234.s009.docx]

**The Editor**

**PLOS Medicine**

Dear Editor,

We thank the editors and reviewers for their thorough and insightful comments on our manuscript. We found these comments extremely helpful in improving our manuscript. Please see our point by point response to these comments in bold letters below with line numbers referring to revised manuscript submitted alongside.

Please do not hesitate if you have any follow-on questions or suggestions.

Best wishes,

Sanjay Kinra, on behalf of the co-authors

**Requests from the editors**

Please revise your title according to PLOS Medicine's style. Your title must be nondeclarative and not a question. It should begin with main concept if possible. "Effect of" should be used only if causality can be inferred, i.e., for an RCT. Please place the study design ("A randomized controlled trial," "A retrospective study," "A modelling study," etc.) in the subtitle (ie, after a colon).

**Done. Line 2.**

At this stage, we ask that you include a short, non-technical Author Summary of your research to make findings accessible to a wide audience that includes both scientists and non-scientists. The Author Summary should immediately follow the Abstract in your revised manuscript. This text is subject to editorial change and should be distinct from the scientific abstract. Please see our author guidelines for more information: [https://journals.plos.org/plosmedicine/s/revising-your-manuscript#loc-author-summary](https://protect-eu.mimecast.com/s/ynF1CD8vnClvkRTp9j7c?domain=journals.plos.org)

**Done. Line 71-101.**

Abstract
Abstract Background: Provide the context of why the study is important. The final sentence should clearly state the study question.

**Done. Line 38-40.**

Please combine methods and findings into one section. The last sentence of this section should be a limitation of your methodology

**Done. Line 42. Line 59-61.**

Please briefly mention the previous study in the background

**Done. Line 36-38.**

In the methods and findings section please mention when the study was carried out

**Done. Line 43.**

Please include a conclusions section, summarising the study findings. Please address the study implications without overreaching what can be concluded from the data; the phrase "In this study,we observed ..." may be useful. Please interpret the study based on the results presented in the abstract, emphasizing what is new without overstating your conclusions. Please avoid vague statements such as "these results have major implications for policy/clinical care". Mention only specific implications substantiated by the results. Please avoid assertions of primacy ("We report for the first time....") Please remove the interpretation subsection.

**Done. Line 63-69.**

Introduction
References- please place a full stop after the square brackets. For example : rural to urban migration [7,8].

**Done. Throughout.**

“…notably childhood home
environment (including parental behaviours) and behaviours, and to a lesser extent
genetics”. I think this needs to be clarified as genetics do play a strong role in adiposity and there is emerging evidence to suggest that it plays a role in diet/physical activity too

**Done. The sentence now reads, ‘However, in such comparisons, the role of unmeasured confounding due to differences in pre-migration characteristics of urban migrants, such as genetics, childhood home environment (including parental behaviours) and behaviours in adolescence should not be ignored.’ Line 125-128.**

Please provide the dates of the Indian Migration study here.

**Done. Line 132.**

Please clarify this sentence “Based on data from elsewhere, we hypothesised that the greater adiposity in urban migrants was primarily due to the differences in their physical activity”

**Done. The sentence now reads, ‘Based on findings from other LMICs noted above (e.g. China, Peru and Guatemala), we hypothesised that the greater adiposity in urban migrants was primarily due to a reduction in their energy expenditure.’ Line 142-144.**

Please clearly state the hypotheses being tested in this study and outline the aims.

**Done. The sentence now reads,** **‘In this study, our primary aim was to investigate the relative contribution of energy intake and expenditure to excess adiposity in urban migrants. Our secondary aim was to identify specific dietary and physical activity behaviours contributing to this excess adiposity.** **Based on findings from other LMICs noted above (e.g. China, Peru and Guatemala), we hypothesised that the greater adiposity in urban migrants was primarily due to a reduction in their energy expenditure.’ Line 139-144.**

Please clarify why the study results were not published sooner-since migration, diet and behaviours have changed since the original study was carried out. This information should be provided in the methods and or discussion sections.

**Done. In discussion, states,** ‘**The present analysis was not conducted earlier as this was not one of the original hypotheses.’ Line 405-406.**

Methods
Please clarify if the nutrient databases are available online and provide a citation.

**Yes. Citations now provided. Line 170.**

Please provide a list of the open-ended questions used to determine physical activities specific to participants.

**Done. We have now provided this, adding ‘e.g. ‘apart from work, how do you spend your time (over last one month): i) Sports/games/exercise (for example walking, badminton, jogging, cricket….etc).’ The exceptions to this were for sleep, standing, sitting, walking at work, and ‘other’ activities which were closed questions, e.g. ‘on average how many hours in a day do you sleep?’ Line 187-190.**

Please cite the individual guidelines used to determine diet and physical activity levels in participants.

**Done. Line 196, Line 201. Line 206.**

Please expand “appropriate adjustments were made using standard methods” citing the methods used.

**Done. ‘If this value was < 24 hours, a residual time variable was generated and a standard MET value of 1.4 was applied, whereas if this value was more than 24 hours, duration of each individual activity was reduced proportional to the amount over-reported [27]. For occupational activities more strenuous than walking, the Integrated Energy Index was applied, to correct for unreported rests which occur in these activities’ Line 198-201.**

Has the definition of “urban” changed since this study was completed? Please mention this in the discussion section as a potential limitation. Same goes for any potential recall bias in diet and physical activity measurements.

**The definition of urban (total population, density, and % employed in agricultural occupations) has not changed since the study was completed****. It is possible that a few of the 1000+ study villages would now be classed as urban, but this would be unlikely to have a major impact on the study findings. We have now mentioned this as a limitation in the discussion section (Line 413-415). We have also acknowledged potential bias in diet and activity measurements as a study limitation (Line 397-398).**

Please clarify the time points at which skin fold thickness was measured.

**This was a cross-sectional study and all measurements, including skinfolds, were measured only once at the same time as the rest of the measurements.** **We have added the sentence, ‘Sibling pairs were examined together and all data, including measurements of skinfold thickness, were collected at the same time (i.e. cross-sectional study design).’ Line 156-158.**

Please provide the name(s) of the institutional review board(s) that provided ethical approval.

**Done. Line 253-254.**

Please report the study according to STROBE guidelines, providing a completed STROBE checklist as supplementary information. Please mention at the beginning of the methods section that the study has been reported in accordance with STROBE guideline and the checklist is provided as SI file xx

**Done. Line 148.**

Please present and organize the Discussion as follows: a short, clear summary of the article's findings; what the study adds to existing research and where and why the results may differ from previous research; strengths and limitations of the study; implications and next steps for research, clinical practice, and/or public policy; one-paragraph conclusion.

**Done. Line 325-450.**

“increasing physical activity in urban areas (e.g. improving walkability of streets and
green spaces)” could there be challenges to this? Increasing pollution levels or the rapid urbanisation of areas causing a decrease in green spaces? Please discuss

**Done. Line 421-426.**

**Comments from the reviewers:**

**Reviewer #1:** I confine my remarks to statistical aspects of this paper. Unfortunately, I think there are some problems with the analysis

The authors need to add standard of living to the regression. Urban dwellers had much higher standard of living and this is surely related to diet (and probably to exercise and sedentary time).

**We agree that the changes in diet and activity are likely due to improvements in the standard of living of urban migrants. However, we believe that our analysis is appropriate for our study aim, which was to investigate the relative contribution of changes in diet and activity to obesity in urban migrants (regardless of the underlying mechanisms for such changes). Adding standard of living to the regression would address a different study aim (i.e. contribution of changes in diet and activity to obesity among those whose standard of living did not change on urban migration), which would have limited policy relevance in India (most people in India improve their economic circumstances after urban migration). Therefore, we have not made this change but would be happy to receive further advice from the editors and this reviewer.**

In the figures, it is clear that there are high leverage points (which inflate R^2) and outliers (which can change the coefficients.

**Outliers were included in the figures earlier to describe the raw data; however, these were not included in the regression analyses. We have now revised the figures by excluding the outliers (Fig 1a, Fig 1b)**.

I would not change all the variables into percents. I would leave the DV as "difference in % body fat" (not divide by the urban dweller) and leave the IVs as differences in grams per day (or minutes per day) rather than %s. This would be easier to interpret.

**We had analysed the variables as percents in order to make them directly comparable to each other (by having them on the same scale). However, we agree that it makes the results less interpretable. Therefore, we have accepted this suggestion and re-analysed the variables in their original scales and included them as results. The main findings are largely unchanged. However, in analysis of our secondary objective, time spent in moderate to vigorous intensity physical activity is now associated with obesity, and not sedentary behaviour (Table 3). This is plausible, and we believe that these revised estimates represent an improvement on the previous version of the analysis. We thank the reviewer for drawing our attention to this important issue.**

How were variables selected for the multivariable models? Why are there different variables in the two models?

**The first model included only total energy intake and expenditure variables (and relevant confounders) to address our primary hypothesis. The second (multivariable) model aimed to establish the relative contribution of diet and physical activity behaviours that have been frequently associated with obesity in literature; all variables for which we had data were included. We have now clarified this. Line 242-244.**

NOTE: Line numbers would have made the review easier.

**Done. Line numbers are now added throughout.**

p. 2 The section about fruits, vegetables etc at the end of results needs to be reworded to include "significant".

**We have now reworded this sentence to include ‘no clear association’ rather than refer to an absolute significance level. Line 57.**

p. 4 Please give numbers for reliability rather than just saying "acceptable"

**Done. Reliability and validity data now added. Line 179-182.**

p. 5 Same comment - give numbers for reliability and validity

**Done. Reliability and validity data now added. Line 208-212.**

BMI - why use BMI when you have the more accurate skinfold measurements?

**We have used percentage body fat estimated from skinfold thickness as the outcome and not BMI. BMI is presented in Table 1 to help readers more familiar with that measure to contextualise the findings.**

p. 7 Please comment on the huge difference in standard of living.

**Most rural to urban migration in India is for economic reasons. There are high levels of poverty and lack of economic opportunity in rural areas. People migrate to urban areas to improve their standard of living and in many cases remit money to their rural families. We have now reported on this in the results. Line 273-274.**

Peter Flom


**Reviewer #2:** Thank you for the opportunity to review the study by Kinra et al., investigating the role of diet and physical activity in increased risk of obesity among rural to urban migrants in India: the Indian Migration Study.

The cross-sectional Indian Migration study has been undertaken between March 2005 and December 2007 and has been described in detail in the paper 'The effect of rural-to-urban migration on obesity and diabetes in India: a cross-sectional study' published by Ebrahim et al. in PLOS Medicine in April 2010. The main hypothesis of the initial paper was that rural-urban migrants would have higher rates of obesity and diabetes than rural non-migrants. The authors report migration into urban areas to be associated with increases in obesity, and amongst others report results showing physical activity to be decreased and fat intake to be increased in the rural-urban sibling migrants. They observe gender differences in some risk factors by place of origin that require further explanation.

The current study by Kinra et al extends the previous cross-sectional analysis addressing the relative contribution of changes in diet and physical activity to risk of obesity in urban migrants. Major results of the paper, i.e. 11% higher energy intake (mainly through fat intake) and 17% lower energy expenditure by physical activity accounting for 2.3% and 6.9% of the difference in adiposity between siblings, add to the results described by Ebrahim and colleagues, yet provide limited additional information to be used for the development of targeted prevention / intervention programmes.

**We believe that our primary study finding that energy intake and expenditure contribute equally to the risk of obesity in urban migrants is extremely informative for policy makers. It suggests that intervention/programs need to be multicomponent and target both diet and physical activity behaviours. Existing interventions in India have often focussed exclusively on diet or physical activity, because of strongly held beliefs among policy makers about the importance of one or the other. In addition, we have identified some specific diet and physical activity targets for such interventions. There are limited data to inform such interventions in India.**

Whilst the initial paper made the case for sex-specific analysis of life-style related factors, the current analysis presents results adjusted for sex rather than sex-specific results, generally considered important for tailored prevention and intervention programmes.

**The initial paper made a case for sex-specific analyses of cardiovascular risk factors (excluding obesity), since migration-associated difference in blood pressure, lipids, fasting blood glucose, and insulin were noted in men, but not women. However, this was not the case for obesity, which showed similar migration-associated differences between men and women. Furthermore, the initial paper had a larger sample size (it also included urban non-migrants and siblings of opposite sex), which allowed sufficient power for sex-stratified analyses. We favoured combined analyses in this paper, after confirming absence of interaction by sex, as it allowed us to robustly investigate our main hypotheses.**

Further, the initial, very well conducted study, has been undertaken 13-15 years ago limiting applicability of results for prevention and intervention efforts within the context of profound life-style changes since 2005 linked to e.g. increasing urbanization and globalization of e.g. food commodities such as availability and affordability of ultra-processed food and possibly other societal factors influencing life-style factors of the general population residing both in rural and urban areas.

**We agree that this is limitation of the study. We have now added the following text to the discussion, ‘The pace of urbanisation and globalisation has increased dramatically over the past decade, and the profound changes in lifestyles and environment could limit the applicability of findings to contemporary populations. There is a great deal of variation in economic development across India (for example, Gross Domestic Product of Delhi is eight times that of the state of Bihar); as a result, the study findings could apply to other areas of India that are at a similar stage of economic development as the study sites at that time. Furthermore, the within-sibling differences in these risk factors may still be generalisable if the societal changes affected urban and rural areas to a similar extent. It is possible that a few of the 1000+ study villages would now be classed as urban, but this would be unlikely to have a major impact on the study findings. Nevertheless, the study findings need to be confirmed in more contemporary populations from multiple settings in India.’ Line 406-417.**

Further comments:
Given the significant difference in age between siblings residing in rural and urban India, presentation of age-adjusted graphs would be preferable to take into account any age-related differences, such as for example the well-known correlation of age with higher percentage of body fat.

**We have used the graphs to display the raw data. Age-adjusted regression coefficients are already shown in Table 2, so doing this would repeat the same information. We are happy to receive further guidance on this.**

In line with the initial paper a discussion on the strengths and limitations of the sibling design as compared to other designs for the study of migration-related changes, the potential bias related to the low response rate, as well as a discussion on the ability of the current study to investigate the effect of short, medium and long-term migration on changes in risk factors and subsequent cardio-metabolic outcomes of rural-urban migrants are warranted.

**Done. Line 361-382.**

**Reviewer #3:** This is a well-designed, interesting and useful study. Also very well written. I have very minor comments below.

The sample is described as geographically representative? Does this mean the proportion of participants from the North, central and Southern India matched the proportion of the Indian population in each of these regions? It would be helpful to understand why this is important- i.e.: that similar patterns were seen in all three regions so in cities throughout India rural-urban migrants are likely to make similar behavioural changes (despite the different cultures in Northern, central and Southern India)?

**Our study was designed to capture broad variation between areas, particularly north and south, as there are important differences in genetics and lifestyles. However, the sample was not representative of India (hence the use of the word ‘geographically representative’). To avoid misunderstanding, we have now deleted ‘geographically representative’ and substituted ‘sample from north, centre and south of India’. There was no evidence for effect modification by centre. We have included this statement in results (Line 295-296) and discussed the need for studies in different regions of India in implications for future research (Line 435-439).**

In the Methods section: "each participant was asked to invite one non-migrant full sibling of the same sex and closest to them in age still residing in their rural place of origin" but then later: "The analyses were restricted to sibling pairs of the same sex to minimise the potential for bias from difference in associations between sexes". And then in the first paragraph of the results there is no suggestion that eligibility required a sibling of the same sex. This is just slightly confusing, I wonder whether the methods should have read "each participant was asked to invite one non-migrant full sibling, *preferably* of the same sex and closest to them in age" (i.e. there were mixed sex pairs in the study but not in this analysis?).

**Yes, that is correct, and we have reworded accordingly. We have now clarified in methods (Line 155-156) and mentioned the number of mixed sex pairs that were in the study but not in this analysis in results (Line 266-268).**

Although the design of this study means that childhood circumstances broadly are similar between the two groups, and genetics also 50% shared- I think it is worth considering how the migrating sibling is often chosen and supported by a family- having the opportunity to migrate and in return sending support back to their rural family (so the family chooses the sibling most likely to thrive after migration). Urban siblings were slightly older (3 years) this suggests the possibility of other systematic differences between the migrant sibling and the rural sibling which might confound the findings. E.g.: despite the same childhood home they may have had different childhood experiences within that home including the often older, migrant sibling getting the lion's share of resources- perhaps means they have different dietary and physical activity behaviours and a different BMI even before migration and these things aren't due to exposure to the urban environment (particularly
noting that years in the urban environment was not significant in the model). Agreed that the lack of difference in height and education is reassuring. I think it would still be worth stating this in the limitations.

**We agree and had mentioned ‘healthy migrant’ bias earlier as a possible limitation. Nevertheless, in view of these comments, we have now expanded on this limitation along the suggested lines.** **Line 372-380.**

In the discussion: "existing evidence on behavioural changes associated with rural to urban migration in LMICs is limited and points to a decline in physical activity as the primary change" also " there are no comparable studies of rural to urban migration from India". In analysis of the WHO-SAGE survey which includes data from India ([https://journals.plos.org/plosone/article?id=10.1371/journal.pone.0122747](https://protect-eu.mimecast.com/s/aNHLCExwoH59NQtRBrXA?domain=journals.plos.org) ) we found the rural to urban migrants achieved significantly less occupational physical activity although more leisure time physical activity and active travel. Although the rural to urban migrants were not siblings of the rural participants so may have different by home environment and genetics, so fair enough to say this was not comparable- but still relevant to this discussion (although I know cheeky to be referring to a paper of which I'm an author).

**As acknowledged by the reviewer, we focussed on studies that had attempted to control for potential confounding inherent in unselected comparison of migrant/urban and rural populations. Furthermore, our focus was on studies that had tried to establish the contribution of diet and activity behaviours to excess obesity in urban migrants, rather than urban/rural differences in diet, activity and obesity, individually. Not all behaviours that were different between urban migrants and rural siblings in our study were found to predict excess adiposity in urban migrants. Nevertheless, given that this study included participants from India, we accept that it merits inclusion in our manuscript. We have now discussed the findings from this study. Line 346-351.**

Any attachments provided with reviews can be seen via the following link:

__________________________________________________
In compliance with data protection regulations, you may request that we remove your personal registration details at any time. (Use the following URL: [https://www.editorialmanager.com/pmedicine/login.asp?a=r](https://protect-eu.mimecast.com/s/zr5ICG80qC5YQVt61qDB?domain=editorialmanager.com)). Please contact the publication office if you have any questions.
