# Supplementary material for: Relative contribution of diet and physical activity to increased adiposity among rural to urban migrants in India: A cross-sectional study
Source: PLoS Med. 2020 Aug 7;17(8):e1003234. doi: 10.1371/journal.pmed.1003234 (PMC7413404; doi:10.1371/journal.pmed.1003234)
Supplement: Attachment — Submitted filename: Response to editors comments_Kinra et al_17 june 20.docx [file pmed.1003234.s010.docx]

**The Editor**

**PLOS Medicine**

Dear Editor,

Thank you for further comments on our revision. Please see our point by point response to these comments in bold letters below.

Please do not hesitate if you have any follow-on questions or suggestions.

Best wishes,

Sanjay Kinra, on behalf of the co-authors

-------------------------------------------------------------------------------------

**Requests from Editors**

I note the questionnaire is cited, but if possible please include here as a Supp file.
**Done as S1 Text.**

Please ensure the STROBE uses sections and paragraph numbers instead of lines as these wont be in the final version. also page numbers change on formatting, so please avoid those too.

**Done.**

Age of data - it is noted that the data are old. can this be updated or is it possible to comment on in the MS?

**This has already been done in response to one of the earlier comments. The relevant sentences from the Discussion (last paragraph of Strengths and Weaknesses section) are pasted below. We hope that they cover this issue, but please let us know if not.**

**‘The study was carried out over a decade ago. The present analysis was not conducted earlier as this was not one of the original hypotheses. The pace of urbanisation and globalisation has increased dramatically over the past decade, and the profound changes in lifestyles and environment could limit the applicability of findings to contemporary populations.’**

**Comments from Reviewers:**

**Reviewer #1:** The authors have addressed my concerns and I now recommend publication

**Thank you.**

**Reviewer #2:** Thank you for responding in detail to the initial review comments. Yet, I do still doubt whether the current paper adds significantly to the original paper from Shah Ebrahim et al who already addressed the relative contribution of changes in diet and physical activity to risk of obesity in urban migrants.

Further, I consider sex-specific analysis of major importance as in most contexts, including in India, dietary behaviour and physical activity patterns are gendered, so that information specific to women, respectively men for the purpose of the development and implementation of tailored prevention and intervention programmes is warranted.

**We agree with the reviewer on the importance of sex-specific analysis, but unfortunately our sample lacked power for this analysis. Nonetheless, we now present sex-specific analyses in supporting information (S4 Table, S5 Table) and have added the following sentence to the results section (final sentence).**

**‘There was no evidence for interaction by study centre or sex (all p>0.1).** **The associations between men and women were similar, although we had limited power to discern sex-specific differences (S4 Table. S5 Table).’**

**Reviewer #3:** The authors have addressed all my comments appropriately and I am happy with this draft of the paper.

Thanks also for the opportunity to read the comments from the editor and other two reviewers. I think the authors have also done a good job of responding to these points.

**Thank you.**

In terms of reviewer 1 "The authors need to add standard of living to the regression. Urban dwellers had much higher standard of living and this is surely related to diet (and probably to exercise and sedentary time)." Just to say, I agree with the authors, that adding standard of living to the regression would examine a different question than the one they have asked. Having read reviewer 1's comments though, I do wonder whether it is worth it (in addition to the results they have presented) because it would allow them to infer what the contribution of *urbanisation* rather than *increased standard of living*- i.e.: if rural populations had a similar increase of wealth would we expect similar changes in diet, physical activity and hence obesity or not? I.e.: What is the contribution of the *urban environment* independent of the *standard of living* changes. It might be particularly interesting if the association between energy intake or energy expenditure and obesity, but not both, were extinguished.

**We have conducted the relevant analyses and found that associations were markedly attenuated except for a weak association between physical activity and obesity. We agree that this finding makes it intriguing. Nevertheless, given the challenges of measuring social conditions accurately, interpreting results from adjustments of variables which are potentially on the causal pathway, and the low study power (most urban migrants had improved standard of living), we are cautious about making too much of these results. Therefore, we propose presenting these data as supplementary information, and have added the following sentences to the main text. We hope that you agree with this approach, but please let us know if not.**

**Results (second last line): “Adjustment for standard of living index markedly attenuated the associations, and only a weak association between physical activity energy expenditure and obesity remained (S2 Table, S3 Table).”**

**Discussion (Implications and further research section): “****An important question for policy makers is the role of physical built environment [35]. In our exploratory analyses, there was a weak suggestion that urban environment may contribute to increased risk of obesity through a decline in physical activity, independent of improvement in personal social conditions. These results should be interpreted with caution due to challenges in measuring social conditions comparably across urban and rural areas of India and the low statistical power of our study to investigate these differences (vast majority of urban migrants had a higher standard of living index compared to their rural siblings). Nevertheless, these findings are intriguing in the context of globalisation and changing social conditions in both urban and rural areas and warrant further research.”**
